# Using Computer Tablets to Improve Moods for Older Adults With Dementia and Interactions With Their Caregivers: Pilot Intervention Study

**DOI:** 10.2196/14530

**Published:** 2019-09-03

**Authors:** Aaron Gilson, Debby Dodds, Arveen Kaur, Michael Potteiger, James H Ford II

**Affiliations:** 1 University of Wisconsin-Madison School of Pharmacy Madison, WI United States; 2 Generation Connect York, PA United States

**Keywords:** mood change, caregiver interactions, older adults, Alzheimer disease, dementia, computer tablets, person-centered care

## Abstract

**Background:**

Persons living with dementia represent a significant and growing segment of the older adult (aged 65 years and older) population. They are often challenged expressively and may experience difficulties with sharing their feelings or moods. Availability of, and easy access to, tablets facilitates the use of information and communication technologies (ICTs) as a delivery mechanism for nonpharmacological interventions, especially for persons living with dementia. Evidence of the impact of ICTs in different community settings on mood with older adults and the impact of engagement on their caregivers is needed to promote broader adoption and sustainment of these technologies in the United States.

**Objective:**

This study aimed to determine the extent of the effects of tablets on positive mood change and examine the effects of study variables on care recipients’ mood changes and caregivers’ daily interactions.

**Methods:**

The tablet intervention was developed and evaluated in five programs. The primary outcome was caregivers’ assessment of care recipients’ mood (n=1089) before and after a tablet engagement session using an eight-point mood visual analog scale. Session influence on caregivers’ daily activities was captured for a subsample of participants (n=542). Frequency distributions were computed for each study variables. Chi-square tests of association were calculated to determine the association of the variables on mood changes for all care recipients, as well as those being treated in skilled nursing facilities and in-home, and then for those that affected caregivers’ daily activities.

**Results:**

The study sample comprised 1089 care recipient and caregiver engagement sessions. Cumulatively, 50.78% (553/1089) of care recipients showed a transition from negative to positive moods, whereas another 41.78% (455/1089) maintained an already-positive mood after the caregiver engagement session. Chi-square analyses demonstrated that positive mood changes resulted from using music (χ^2^_10_=72.9; *P*<.001), using YouTube as the sole app (χ^2^_12_=64.5; *P*<.001), using multiple engagement strategies (χ^2^_2_=42.8; *P*<.001), and when cared for in a skilled nursing facility (χ^2^_4_=236.8; *P*<.001) across the entire care recipient sample. In addition, although many features of the engagement session positively influenced the caregivers’ day, the largest effect was observed when care recipients’ mood was considered to have improved following the session (χ^2^_4_=234.7; *P*<.001).

**Conclusions:**

The study is one of the first in the United States to explore the impact of ICTs, in particular managed tablets and Web-based video services that can be used on a tablet through an app, on improving mood in persons living with dementia, and enhancing caregivers’ perceptions about their care recipient interactions. Importantly, these pilot data substantiate ICTs as part of a personalized engagement approach, as beneficial alternatives to pharmaceutical interventions for mood enhancement. However, a more comprehensive study that explores the ICT’s impact on additional clinical outcomes is needed to confirm these preliminary findings.

## Introduction

### Background

The number of people living with Alzheimer and related dementia is projected to triple by 2050 [1-3]. Persons living with dementia can have complex physical health concerns, comorbid medical conditions, and exhibit behavioral and psychological symptoms of dementia (BPSD) [4-11]. In fact, professional caregivers of persons living with dementia already note that responding to BPSD is one of the greatest care challenges and can negatively affect the health of caregivers [12-14] especially in long-term health care facilities, where two-thirds of residents have dementia [9,13,15,16]. Family caregivers of persons living with dementia are also are at risk for higher rates of depression, health and sleep issues, social isolation, and mortality [14,17].

Pharmacological or nonpharmacological interventions can help manage BPSD in older adults with moderate-to-severe cognitive impairment. Medications may control the physical aspects of BPSD but can have many side effects [18]. Nonpharmacological approaches, such as reminiscence therapy, music therapy, or behavior management techniques, are preferred because they sustain cognitive function, improve quality of life, and mitigate BPSD [19-25]. The growing number of persons living with dementia, overall care challenges, and the negative effects of pharmacological interventions combine to highlight the need, as well as the opportunity, for additional nonpharmacological interventions.

Availability of tablets and mobile phones has changed how nonpharmacological interventions can be delivered to older adults. Tablets can enhance residents’ emotions through the use of multisensory activities. Individuals can now obtain informational content, interact socially (including participating in Web-based games), listen to music, or reminisce about the past or a recent special event. Easy access to information and communication technologies (ICTs) such as tablets facilitates the delivery of nonpharmacological interventions especially for persons living with dementia and their caregivers [26-28]. For persons living with dementia, ICTs such as YouTube, a Web-based video service, can be used to deliver reminiscence therapy interventions on a tablet through an app [29]. For these individuals, the use of ICTs mitigates motor and sensory impairments; compensates for memory deficits; or enhances latent skills and abilities for sensory awareness, musical responsiveness, and emotional memory [30,31]. Overall, the use of ICTs to deliver nonpharmacological interventions for persons living with dementia benefits care recipients’ well-being and mood; communication and interactions; and caregivers’ mental health (eg, depression and anxiety), self-efficacy, and relationship with the care recipient [26,28,29,32-39]. An additional benefit is the potential for persons living with dementia to remain in the home for as long as possible. This outcome is a widespread aspiration, with “Nearly 90 percent of people over age 65 [wanting] to stay in their homes for as long as possible” [40]. Therapeutic approaches that help persons living with dementia live at home longer also reduces overall health care costs [41].

### Development of a Tablet Intervention

Although successful use of tablets originated and empirically documented about a decade ago in Great Britain, with investigations continuing into the present both in Europe and beyond [42-47], its adoption in the United States has been slow [34,48-55]. Two Great Britain studies offer a progressive look at how tablets can be applied to improve communications between persons living with dementia and their caregivers [56,57]. The first study compared a traditional reminiscence programs (noncomputer) with computerized touchscreen, one-on-one, reminiscence sessions and tested the hypothesis that caregivers offer greater choices and engage in more conversational activities during the touchscreen sessions than during the traditional one-on-one reminiscence sessions with physical properties [56]. After comparing the 2 types of sessions across both verbal and nonverbal parameters, such as laughter, singing, pointing, and eye gaze, the touchscreen system was shown to provide greater benefits for both caregivers and persons living with dementia. The second study was based on the central concept that “external memory aids have demonstrated a reduction in resistance to care...” [57], and using the iPad as the focus of joint attention not only relieves some of the burden of communication for caregivers but also provides the opportunity for the caregiver to support greater independence in persons living with dementia through scaffolding behavior.

The project described in this study extends this seminal international work to implement a tablet intervention using multiple device management and software apps in different community settings and document the effect on mood with older adults, as well as the impact of engagement on their caregivers. This manuscript reports aggregate findings from a pilot study evaluating the impact of personalized tablet engagement sessions. Four project aims represent the purpose of this study to examine study variables effects on mood changes in (1) the overall care recipient sample, (2) the subsample of care recipients being treated in skilled nursing facilities (SNFs), (3) care recipients in home care only, and (4) caregivers’ daily activities.

## Methods

### Personalized Tablet Engagement

One of the primary advantages in using a tablet for engagement activities for persons living with dementia is the ability to personalize activities because every person has a unique lifetime of experiences. For this program, the term *personalized tablet engagement* reflects a tablet engagement session that is designed by a caregiver based on the personal history of the care recipient. The session is presented to the care recipient, who is given the option of participating. In this way, tablet engagement is both based on an individual’s interests, abilities, and preferences and allows the individual to exercise autonomy.

### Study Settings

Since 2015, Generation Connect initiated partnerships to evaluate the effectiveness of tablet interventions in dyads of caregivers and persons living with dementia. The Music & Memory Foundation used tablet computers that were preconfigured with recommended apps, deployed with management software, and implemented with training resources created and facilitated by Generation Connect. These tablets allowed tailored interactions to residents’ interests or hobbies to expedite engagement and enhance emotions through multisensory activities.

This study describes a naturalistic study in which the Music & Memory Foundation and a Visiting Angels in-home care agency franchise collaborated with Generation Connect to develop and conduct an ongoing tablet engagement pilot study involving 5 different programs across time (2017-2018). Multimedia Appendix 1 describes the 5 programs contributing the tablet engagement session data analyzed for this study. 

### Participants’ Attributes

The intervention was designed to be useful for persons living with dementia, and all participating communities opted into the pilot study and self-selected the residents that participated, no standardized test was used to record non-normative memory loss. The sessions were not limited restricted to a dementia population and contained a variety of other conditions as well. However, participants’ demographics beyond these primary challenges were not collected in the pilot programs.

### Data Collection

#### Caregiver Training for Tablet Engagement Sessions

Caregivers were trained to gather personal history and take personal lifestyle into consideration when selecting the activities for care recipients. As a result, caregivers were better able to provide multisensory tablet engagement for specific and prior positive life events, such as reminiscing or listening to personal music. All training was conducted by members of Generation Connect in a series of live webinars, Web-based courses, and printable handouts. Instructors demonstrated apps and talked through best practices for meeting the engagement training elements of discovery, engagement, and planning, emphasizing that the engagement process is guided by care recipient needs (see Multimedia Appendix 2). The training process was dynamic and changed over the course of time as new information was gathered.

#### Engagement

##### Personalized Tablet Engagement

Many older adults are hesitant to interact with a device or computer but are comfortable watching videos. The caregiver’s goal for engagement is to *make the tablet disappear* and provide an enjoyable personalized experience. The most successful engagement sessions reflect a *best friend* approach, and engagement is based on mentoring not care recipient mastery. This study did not provide official tracking of session frequencies or lengths.

##### Selection of Apps

This project was conceptualized over a number of years, based on supporting research at that time. As a result, apps available in the public domain (see Multimedia Appendix 3) were chosen because they represented at least 1 of 4 basic types of traditional activity-based engagement activities that evidence suggests can be beneficial for people with dementia: reminiscing, music, images, and games [58-63].

##### Engagement Session Feedback Survey

Data collection in the programs was to improve residents’ socialization, personalized care, and mood management through nonpharmacological interventions and make the day better for caregivers. The short, 5-question survey was available in a Web app on the tablets’ homepage for easy caregiver access (see Multimedia Appendix 4). Instructors demonstrated how to use the app survey to record session feedback information.

#### Mood Rating

**Figure 1:**
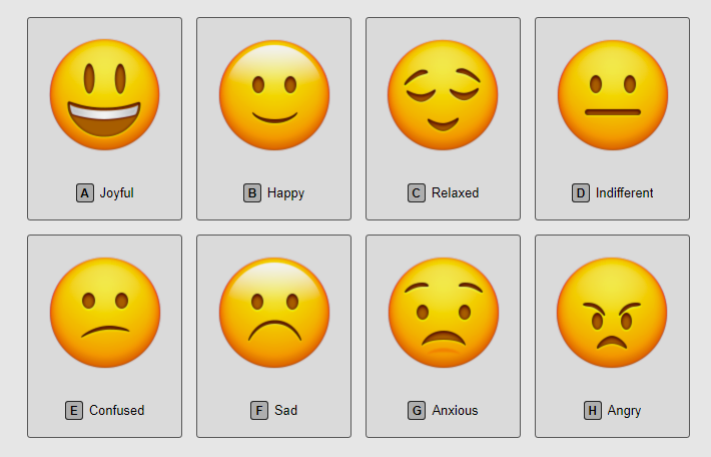
Participants’ mood rating scale.

Persons living with dementia are often challenged expressively and may experience difficulties with sharing their feelings or moods. A visual analog mood scale (VAMS) represents a valid tool to assess baseline and changes in mood, especially in those with expressive disabilities [48,52,55] and for dementia [64]. VAMS consists of a sequence of 8 faces (Figure 1) and was incorporated into the tablets used by Generation Connect. Caregivers reported changes in care recipient mood before and immediately after their tablet engagement activity.

For this analysis, individual moods were categorized into 2 domains: (1) negative moods (ie, angry, anxious, sad, confused, and indifferent) and (2) positive moods (ie, relaxed, happy, and joyful). *Indifference* was considered a negative mood because of its potential to adversely affect other aspects of the care recipients’ functioning throughout the day, as well as influence caregivers’ activities [65]. Mood changes were then categorized according to the mood assessed before the care recipient and caregiver engagement compared with the mood observed after engagement, resulting in 4 categories: worsening of mood (ie, transitions from positive to negative mood domains), maintaining negative mood (ie, changes remain within the negative mood domain), maintaining positive mood (ie, changes remain within the positive mood domain), or improvement of mood (ie, transitions from negative to positive mood domains).

#### Caregivers’ Daily Activities

The impact of the sessions with older adults was captured for a subsample of caregivers across the 5 programs. Caregivers responded to the following question: how did the engagement session impact your day? Impact was rated using a 5-point scale anchored at 0 (made it worse), 3 (no impact), and 5 (made it better). Scale values for *2* and *4* had no assigned anchor label but were, in this analysis, assigned *slight worsening* and *slight improvement*, respectively.

### Data Cleaning

All data were reviewed for accuracy and completeness. In addition, data cleaning and label standardization ensured response uniformity before analysis. Our team then reviewed and converted the variables into the following categories:

- Care recipients’ primary physical challenge—memory loss, nonambulatory, movement disorder, communicative or expressive disorders, or other
- Method used for care recipient and caregiver engagement (either singularly or combined with other methods)—music, stories video, games, communication, photos, or other
- Type of strategy of care recipient and caregiver engagement (either singularly or combined with other strategies)—music, reminiscing, socialization, relaxation, or achievement
- Number of strategies used—either 1 or 2 or more
- Type of app used to facilitate strategies—YouTube only, Personal playlist only, Personal photos or videos only, Google only, Puzzles only, combination of apps, or other
- Number of apps used to facilitate strategies—either 1 or 2 or more

Facility type was self-reported as *skilled nursing*, *home care*, or *other* (which consist of Continuing Care Center, Assisted Living Facility, Long-Term Care Facility, and Veterans Hospital). Facility locations were classified as rural or urban using the US Department of Agriculture Rural-Urban Continuum Code, and for Canadian facilities, rural or urban status was determined through website search.

### Statistical Analysis

Frequency distributions were computed for each of the study variables, including the type of care recipients’ mood changes. All variables were collected as categorical and ranged from dichotomous, trichotomous, up to 6 categories. Chi-square tests of association were calculated to determine the influence of the variables on mood changes for all care recipients, as well as those being treated in SNFs and in-home, and then those that affected caregivers’ daily activities.

### Ethics Consideration

The project was determined to constitute quality improvement or program evaluation and in accordance with federal regulations, the project did not constitute research as defined under 45 Code of Federal Regulations 46.102(d).

## Results

### Posthoc Power Analysis

Posthoc power analysis for the chi-square tests demonstrated superior power for all analyses. The power levels calculated for all 4 project aims conformed to Cohen’s accepted standard of a four-to-one weighting between type II and type I error risks [66]. In addition, no significant chi-square results were associated with more than 33% of contingency table cells having expected counts of less than 5.

### Descriptive Statistics

Frequencies were computed for each study variable and each of the analyzed samples (ie, full sample, SNFs only, and home care only), as shown in Multimedia Appendix 5, which also demonstrates a relatively unequal distribution of resident mood changes after use of the tablet.

Table 1 represents overall mood improvement after the use of the app technology among individuals with various disabilities and is further illustrated when examining frequencies within each of the mood change categories.

**Table 1:** Mood transitions for individuals.

Mood before session	Mood after session, n (%)							
	Angry	Anxious	Sad	Confused	Indifferent	Relaxed	Happy	Joyful
Angry (n=33)	2 (6)^a^	—^b^	—	—	—	2 (6)^c^	11 (33)^c^	18 (55)^c^
Anxious (n=52)	—	4 (8)^a^	2 (4)^a^	2 (4)^a^	4 (8)^a^	24 (46)^c^	11 (21)^c^	5 (10)^c^
Sad (n=64)	—	—	2 (3)^a^	1 (2)^a^	—	3 (5)^c^	30 (47)^c^	28 (44)^c^
Confused (n=70)	—	1 (1)^a^	—	3 (4)^a^	6 (9)^a^	29 (41)^c^	23 (33)^c^	8 (11.4)^c^
Indifferent (n=407)	1 (0.2)^a^	1 (0.2)^a^	—	7 (1.7)^a^	36 (8.9)^a^	91 (22.5)^c^	176 (43.2)^c^	95 (23.3)^c^
Relaxed (n=119)	—	—	—	1 (0.8)^d^	6 (5.0)^d^	28 (23.5)^e^	70 (58.8)^e^	14 (11.8)^e^
Happy (n=326)	—	—	—	—	—	7 (2.1)^e^	257 (78.8)^e^	62 (19.0)^e^
Joyful (n=18)	—	—	1 (6)^d^	—	—	—	5 (28)^e^	12 (67)^e^

^a^Not applicable as no care recipients transitioned to this mood from their beginning mood.

^b^Maintaining negative mood.

^c^Transition from negative to positive mood.

^d^Worsening of mood.

^e^Maintaining positive mood.

#### Worsening of Mood (Transition From Positive to Negative Mood Domains; n=8)

Any worsening of mood represented the mood change category with the fewest number of care recipients. One care recipient began an engagement session in a joyful mood but ended up confused after the tablet intervention, whereas another care recipient transitioned from relaxed to confused. The remaining 6 cases involved a transition from relaxed to indifferent.

#### Maintaining Negative Mood (n=72)

Within this category, 65% (47/72) of care recipients showed no mood change, and most (n=36) of these moods were viewed as indifference. The most frequent mood changes were found for transitions from indifferent to confused (n=7) and confused to indifferent (n=6).

#### Maintaining Positive Mood (n=455)

Within this category, 65.3% (297/455) of care recipients showed no mood change, and most (257/297, 86.5%) of these moods were viewed as happy. The most frequent mood transitions within the positive mood domain represented changes from relaxed to happy (n=70) and then from happy to joyful (n=62), and the most notable improvement was found for transitions from relaxed to joyful (n=14).

#### Improvement of Mood (Transition From Negative to Positive Mood Domains; n=553)

Two-thirds (362/553) of all care recipients began the sessions in an indifferent mood and then increased to relaxed (n=91), happy (n=176), or joyful (n=95). Furthermore, the proportion of care recipients achieving joyful mood after the session was noteworthy. Cumulatively, 21% of care recipients who began the session as either anxious (5/52) or confused (8/70) became joyful as a result of the session, whereas 44% (28/64) of care recipients who were sad ended the session in a joyful mood. Importantly, 18 of the 33 care recipients (58%) transitioned from being angry to being joyful, demonstrating the greatest movement possible within this mood category.

### Chi-Square Analyses

Table 2 contains the frequencies for each study variable within the 4 separate mood change categories, which provides insight into the category of each variable that contributed to the mood effect.

**Table 2:** Chi-square comparisons of variables to mood change in full sample.

Variables			Mood change		
			Worse or maintaining negative mood (n=80), n (%)	Maintaining positive mood (n=455), n (%)	Mood improvement (n=553), n (%)
**Primary challenge^a^**					
	Memory loss		61 (5.60)	424 (38.93)	300 (27.55)
	Nonambulatory		6 (0.55)	6 (0.55)	93 (8.54)
	Movement disorder		10 (0.92)	13 (1.19)	105 (9.64)
	Communicative or expressive disorders		2 (0.18)	9 (0.83)	45 (4.13)
	Other		1 (0.09)	4 (0.37)	10 (0.92)
**Engagement method use (singularly or one of multiple)^b^**					
	Music		22 (2.3)	127 (13.4)	228 (24.1)
	Stories video		12 (1.3)	101 (10.7)	86 (9.1)
	Games		13 (1.4)	67 (7.1)	33 (3.5)
	Communication		1 (0.1)	47 (5.0)	48 (5.1)
	Photos		3 (0.3)	48 (5.1)	24 (2.5)
	Other		6 (0.6)	21 (2.2	59 (6.2)
**Type of strategies used^c^**					
	**Music**				
		Singularly	15 (2.9)	89 (17.3)	95 (18.5)
		One or more	26 (5.1)	81 (15.8)	207 (40.4)
	**Reminiscing**				
		Singularly	2 (0.7)	5 (1.6)	36 (11.8)
		One or more	7 (2.3)	93 (30.6)	161 (53.0)
	**Socialization**				
		Singularly	9 (1.7)	119 (22.2)	58 (10.8)
		One or more	15 (2.8)	129 (24.07)	206 (38.4)
	**Relaxation**				
		Singularly	2 (0.8)	4 (1.7)	7 (3.0)
		One or more	21 (8.9)	60 (25.4)	142 (60.2)
	**Achievement**				
		Singularly	10 (4.0)	60 (23.9)	33 (13.1)
		One or more	6 (2.4)	62 (24.7)	80 (31.9)
**Number of strategies used^d^**					
	1		40 (3.75)	276 (25.89)	225 (21.11)
	≥2		33 (3.10)	170 (15.95)	322 (30.21)
**Type of app used for strategies^d^**					
	YouTube only		10 (0.94)	117 (10.98)	178 (16.70)
	Personal Playlist only		36 (3.38)	115 (10.798)	157 (14.73)
	Personal photos or videos only		0 (0.00)	35 (3.28)	41 (3.85)
	Google only		3 (0.28)	53 (4.97)	35 (3.28)
	Puzzles only		6 (0.56)	48 (4.50)	24 (2.25)
	Combinations of apps		10 (0.94)	52 (4.88)	45 (4.22)
	Other		11 (1.03)	27 (2.53)	63 (5.91)
**Number of apps used for strategies^e^**					
	1		66 (6.20)	394 (37.00)	498 (46.76)
	≥2		10 (0.94)	52 (4.88)	45 (4.23)
**Urban or rural status^a^**					
	Rural		39 (3.58)	64 (5.88)	306 (28.10)
	Urban		41 (3.76)	392 (36.00)	247 (22.68)
**Facility type^a^**					
	Skilled nursing		28 (2.57)	42 (3.86)	303 (27.82)
	Home care		46 (4.22)	393 (36.09)	227 (20.84)
	Other		6 (0.55)	21 (1.93)	23 (2.11)

^a^Percentages for primary challenge in the chi-square analysis are based on a sample of 1089 care recipients. Differences as compared with the total sample is based on missing data, which were excluded from the chi-square analysis.

^b^Percentages for engagement method use in the chi-square analysis is based on a sample of 946 care recipients.

^c^Percentages for type of strategy used in the chi-square analysis varied based on the strategy. The sample sizes are music (n=513), reminiscing (n=304), socialization (n=506), relaxation (n=236), and achievement (n=251), respectively.

^d^Percentages for number of strategies used and the type of app used for the strategies in the chi-square analysis is based on a sample of 1066 care recipients.

^e^Percentages for number of apps used for strategies in the chi-square analysis is based on a sample of 1065 care recipients.

#### Project Aim 1: Analysis of Full Sample

Chi-square analyses of all care recipients were conducted to determine the extent that significant mood changes occurred for those receiving the intervention (n=1089). When examining the effects of the care recipients’ primary health care challenges on their observed mood, memory loss was associated with maintaining positive mood (χ^2^_8_=191.5; *P*<.001). Engagement methods demonstrated some effect on care recipients’ mood, with music being more likely involved in care recipients’ improvements from negative to positive moods (χ^2^_10_=72.9; *P*<.001). Characteristics of the apps used to engage also influenced mood. When using YouTube as the sole app, care recipients’ mood transitioned from negative to positive after the engagement sessions (χ^2^_12_=64.5; *P*<.001). Results also suggested that some strategies implemented to evoke mood changes were effective. Specifically, using music as a single method (χ^2^_2_=20.1; *P*<.001) or reminiscing (χ^2^_2_=9.9; *P*=.007) as a component of additional strategies was associated with maintenance of positive mood; socialization (χ^2^_2_=38.3; *P*<.001) and achievement (χ^2^_2_=12.9; *P*=.002), when used as a component, were related to care recipients having improved mood. These findings support the separate analysis showing that care recipients using multiple strategies were more likely to be associated with negative to positive mood transitions (χ^2^_2_=42.8; *P*<.001). Examining the influences of facility characteristics revealed that care recipient placement in an SNF was linked to changes from negative to positive moods following engagement (χ^2^_4_=236.8; *P*<.001), whereas placement in an urban setting was associated with maintaining positive mood (χ^2^_2_=186.4; *P*<.001).

#### Project Aim 2: Analysis of Care Recipients in Skilled Nursing Facilities

Subsample analyses were designed to reveal the effects of the study variables on mood changes for care recipients from SNFs only (n=373). Notably, fewer significant results emerged from this sample compared with either the full sample or the home care subsample analyses; however, these results demonstrated a consistent effect on mood. Engagement sessions involving reminiscing were more likely to facilitate care recipients’ going from negative to positive moods as a result of the engagement sessions (χ^2^_4_=12.0; *P*=.02). Socialization, when used as a component of additional strategies, also was related to care recipients having positive mood changes (χ^2^_2_=11.0; *P*=.004). Overall, care recipients using multiple strategies were associated with improvements from negative to positive moods (χ^2^_2_=8.5; *P*=.014). This trend substantiates the finding derived from the full sample, employing 2 or more strategies is an effective approach for positive moods. Finally, engagement sessions occurring in SNFs operating in an urban setting was associated with maintaining positive mood (χ^2^_2_=18.4; *P*<.001).

#### Project Aim 3: Analysis of Care Recipients in Home Care

A subsample analysis also was conducted for care recipients involved only in in-home care (n=666). Care recipients with a movement disorder as their primary challenge were associated with negative to positive mood changes as a result of the intervention (χ^2^_2_=115.7; *P*<.001). Engagement sessions involving music were more likely linked to care recipients’ improvements from negative to positive moods (χ^2^_10_=112.7; *P*<.001). Furthermore, using combinations of strategies involving music was associated with mood improvement (χ^2^_2_=34.4; *P*<.001). The only other strategy achieving significance was the sole use of socialization, which was shown to maintain positive mood (χ^2^_2_=45.5; *P*<.001). Overall, although, any strategy used in combination with others was related to sustaining positive moods (χ^2^_1_=75.8; *P*<.001). When considering specific apps, Personal Playlist, as the sole app, was more likely to be associated with care recipients transitioning from negative to positive mood (χ^2^_12_=135.5; *P*<.001). Finally, home care occurring in urban settings was associated with care recipients sustaining positive mood as a result of the intervention (χ^2^_2_=57.5; *P*<.001).

#### Project Aim 4: Analysis of Caregivers’ Daily Activities

A subsample of caregivers also rated the impact of the intervention on their day (n=542). When care recipients had a movement disorder, the engagement session made the day better for caregivers (χ^2^_2_=10.1; *P*=.006). Music, when used singularly, slightly improved the caregiver’s day (χ^2^_2_=17.2; *P*<.001), whereas socialization as a component of other interventions made the day better for the caregiver (χ^2^_2_=37.2; *P*<.001). Using only 1 strategy (χ^2^_2_=39.4; *P*<.001) or using apps multiple times (χ^2^_2_=12.7; *P*=.002) slightly improved the caregiver’s day, but using only Personal Playlist apps made the caregiver’s day much better (χ^2^_12_=91.9; *P*<.001). In urban settings, the positive impact of the engagement session was only slight (χ^2^_2_=119.8; *P*<.001). When engagement occurred in other than SNFs or home care, the session made the day better for caregivers (χ^2^_4_=11.0; *P*=.03). Importantly, when care recipients’ mood was observed to improve, the engagement session was more likely to make the day better for staff (χ^2^_4_=234.7; *P*<.001).

## Discussion

### Principal Findings

This study is one of the first in the United States to focus on the implementation of tablets across institutional (eg, SNFs) and in-home settings for persons living with dementia. The most apparent implication from this study was the substantial benefit of the tablet interventions to most care recipients. Less than 8% of the entire sample evidenced either the transition of positive to negative mood (8/1089, 0.73%) or the sustainment of negative moods (72/1089, 6.61%) after caregiver engagement sessions. That is, for the vast majority of the entire sample, positive care recipient moods were either maintained or enhanced or were achieved after beginning the session in a negative mood. The unequivocal therapeutic benefit of personalized tablet engagement with music and other approaches seems a function not only of the effectiveness of the particular intervention but also of caregiver commitment to and compassion for the care recipient.

Caregiver supports that maintain interest and participation, as well as promote feelings of achievement and mastery, create a positive experience for participants and caregivers with tablets [33]. Successful interactions should provide achievable goals facilitating feelings of self-worth, which require the caregiver to be *in the moment* and select apps based on resident mood to promote engagement [33]. Study results extend this concept further, indicating that app selection should target multiple strategies (eg, reminiscing or socialization) to facilitate improvements in resident moods.

Our findings also offer guidance around the care recipient and caregiver engagement process. Specifically, the use of a single app, especially YouTube, resulted in moderate to substantial improvements in resident mood across the entire sample. This influence of YouTube supports additional research, indicating that exposure to YouTube videos improves participants’ well-being and mood, as well as their communication, interaction, and engagement [29]. In our study, YouTube was primarily used to share music or stories through video and strategies related to reminiscing, socialization, or music. One resident encounter convincingly illustrates tablet impact on resident mood:

Listened and watched a YouTube video with headphones and closed caption in Navajo. Elder was happy and smiling. He sang along in Navajo and tried [to] teach me the words in Navajo.

From one resident encounter

This session successfully targeted all 3 strategies, and the resident’s mood improved the greatest extent possible, from sad to joyful. Years of experience has shown that YouTube holds great potential for personalization because of the nearly unlimited number of topics available. When given the choice of activity, caregivers are choosing to work with YouTube.

Interestingly, using gaming apps (eg, Puzzable or Solitaire) to engage care recipients did not statistically improve mood, especially compared with other engagement strategies. For these care recipients, the complexity of *playing the game* may have undermined mood improvement. For example, one resident reported the following:

the worst part [of the game] was there were too many questions - wanted too much information before getting to anything interesting.

One resident

Although an unfortunate reaction, this finding supports research suggesting that gaming apps are not as useful for promoting engagement as apps targeting relaxation or telling an individual’s life story in this population [34]. Conversely, other research demonstrates that interactive computer games (eg, a Nintendo Wii) affected participant cognition [67]. These contradictory findings warrant further research to examine the specific gaming features and the interaction of these features with care recipient characteristics and cognitive conditions that prompt mood changes.

Mood changes differed by location. Overall, 82.9% (339/409) of care recipients in rural areas reported more negative moods (sad, confused, angry, anxious, or indifferent) at baseline versus those in urban areas (286/680, 42.1%). Similarly, 88.2% (329/373) of care recipients in SNFs began sessions with a negative mood compared with 40.1% (267/666) of individuals in home care settings. However, after tablet engagement, only 7.5% (28/373) of individuals were assessed with a negative mood. A potential explanation for this finding is a greater continuity of care existing between caregivers and care recipients in home care sessions, including more frequent and sustained interactions. How facility-setting characteristics influence care recipient or caregiver mood and mood changes, along with their feelings of isolation or loneliness, remains crucial research topics when implementing tablets or other ICTs.

### Limitations

Although illustrating numerous areas for investigation, this study had limitations. First, to ease data collection burden on caregivers, no resident or caregiver attributes (such as gender, age, or duration of the primary challenges) or years of caregiving service were documented. Second, although efforts were made to systematize training and data collection, there may have been variability in these processes either across or within programs. For example, the key strategy (eg, music or reminiscing) may have been recorded inconsistently. Third, as caregiver observations determined mood baseline and changes, knowledge of study objectives or varying assessments of residents’ mood could, even unintentionally, introduce bias. Fourth, the time allotted for the care recipient and caregiver interaction may have affected mood changes but was not collected. Finally, findings related to the caregivers’ day suggest that the intervention was beneficial overall, but the effect may differ for larger samples or other care recipient or caregiver samples.

### Conclusions

This study documents further evidence that ICTs, in particular, tablets such as iPads and apps such as YouTube or using a Personal Playlist, improve mood in older adults and enhance caregiver perceptions about their care recipient interactions, especially with persons living with dementia. Importantly, these pilot data substantiate ICTs, as part of a personalized engagement approach, as beneficial alternatives to pharmaceutical interventions for mood enhancement. As was done for this study, matching engagement methods and types of strategies and apps, based on care recipient preferences or other features to maximize their mood effects, is critical for strengthening the utility of this approach. Accumulating evidence and the additional research questions emerging from this nascent examination offer exciting prospects for continued empirical inquiry and bettering care recipient well-being.

## Appendix

Multimedia Appendix 1
Description of tablet engagement programs.

Multimedia Appendix 2
Caregiver training process.

Multimedia Appendix 3
Description of the apps included on the tablet.

Multimedia Appendix 4
Feedback survey questions.

Multimedia Appendix 5
Frequency of study variables.

## Abbreviations

BPSD: behavioral and psychological symptoms of dementia
ICT: information and communication technology
SNF: skilled nursing facility
VAMS: visual analog mood scale
