# Supplementary material for: Using Computer Tablets to Improve Moods for Older Adults With Dementia and Interactions With Their Caregivers: Pilot Intervention Study
Source: JMIR Form Res. 2019 Sep 3;3(3):e14530. doi: 10.2196/14530 (PMC6751094; doi:10.2196/14530)
Supplement: Multimedia Appendix 1 [file formative_v3i3e14530_app1.pdf]

Appendix 1: Description of Tablet Engagement Programs		
**Program Partner and Data Collection Timeframe**	**Care Recipient Population**	**Description**
Consumer Technology Association Grant, Music & Memory Partner Channel<br>(June 2017-August 2017)	Alzheimer's or other forms of dementia<br>cognitive or physical challenges	Funded by a grant from the Consumer Technology Association, for 10 nursing homes throughout the U.S. The purpose of the grant was to learn how tablet technology can be leveraged to enhance mood for elderly and infirm in long-term care. The pilot sought to access which tablet applications are best suited to enhance socialization, cognition, communication, and mood for the aging population living in care facilities. A user guide based on pilot outcomes, entitled Tablet Engagement Enriching Lives with Everyday Technology, was created and published for the Consumer Technology Association. Personalized tablet engagement sessions utilized close to 20 different apps including YouTube, music, images, and games to improve resident moods.
Verizon Grant, Music & Memory Partner Channel<br>(June 2017-August 2017)	Alzheimer's	Funded by a grant from Verizon and ran concurrently with the Consumer Technology Association-funded grant. The goal of this pilot was to offer training and support for care staff to integrate tablets into the daily workings of SNFs. The Verizon grant included 8 6 nursing homes throughout the U.S., and was the only pilot to call out and limit to specific residents with dementia. Personalized tablet engagement sessions consisted of close to 20 different apps including YouTube, music, images, and games to improve resident moods.
Music & Memory Partner Channel Partners<br>(January 2018-March 2018)	Alzheimer's or other forms of dementia<br>cognitive or physical challenges	Involved Music & Memory promoting the Tablet Engagement program through email blasts to their nursing home partner channel. Ten participants were offered a reduced rate that included equipment, online training, live webinars, and support. The goal of the project was to provide staff with new, tablet-based, personalized, non-pharmacologic care and training tools to enable positive mood management for residents, and to improve the care environment for staff. Personalized tablet engagement sessions consisted of utilizing YouTube videos (including music and personal interests such as work, hobbies, or humor) images, and games to improve resident moods.

Visiting Angels<br>(January 2018-March 2018)	Non-normative memory loss<br>cognitive or physical challenges	A Visiting Angels franchise owner invested in the Tablet Engagement pilot for one of their two franchise locations. The purpose of the pilot was to improve personalized, non-pharmacologic, and mood management care through tablet mentoring for clients, and to increase client and staff retention. Personalized tablet engagement sessions consisted of utilizing YouTube videos (including music and personal interests such as work, hobbies, or humor) images, and games to improve resident moods.
Visiting Angels<br>(June 2018-December 2018)	Non-normative memory loss<br>cognitive or physical challenges	The same Visiting Angels home care franchise owner decide to expand tablets for personalized client engagement to a second franchise location. The leadership team identified several clients for the program, all of whom had been known to have dementia. Caregivers received training on best practices, before initiating engagement sessions. Personalized tablet engagement sessions consisted of utilizing music playlists, YouTube videos (including music and personal interests such as work, hobbies, or humor) images, and games to improve resident moods.
