# Supplementary material for: Using Computer Tablets to Improve Moods for Older Adults With Dementia and Interactions With Their Caregivers: Pilot Intervention Study
Source: JMIR Form Res. 2019 Sep 3;3(3):e14530. doi: 10.2196/14530 (PMC6751094; doi:10.2196/14530)
Supplement: Multimedia Appendix 2 [file formative_v3i3e14530_app2.pdf]

Appendix 2: Caregiver Training Process

How Caregivers and Staff Are Trained?

To facilitate the tablet engagement sessions, caregivers completed a combination of online training and a series of in-person or live webinar training. Their training is centered on three elements: discovery, engagement, and planning.

For care community staff, training consisted of three or four live webinars or online courses, with printable handouts. For Visiting Angels staff, the training was conducted live at franchise offices. Both types of training included the following: projection of the iPad onto a screen with instructors demonstrating the apps; a session on best practices for using the apps; a feedback survey app online; and teaching of best practices for using engagement to further safety and security-. Handouts were also available for both groups -. The training process was dynamic and allowed to evolve over time as new information was gathered.

Discovery

The first step in successful personalized tablet engagement is the customization of the session to the individual. Caregivers conduct an initial discovery phase with family members who have a personal history with the care recipient, or with staff members who are familiar with the care recipient's intake information.

For example, one family described their care recipients' fond memories of traveling in Scotland. The care provider created a session using *Google* to view images of the countryside and *YouTube* to take tours and play Scottish music. The following reactions were recorded for the session:

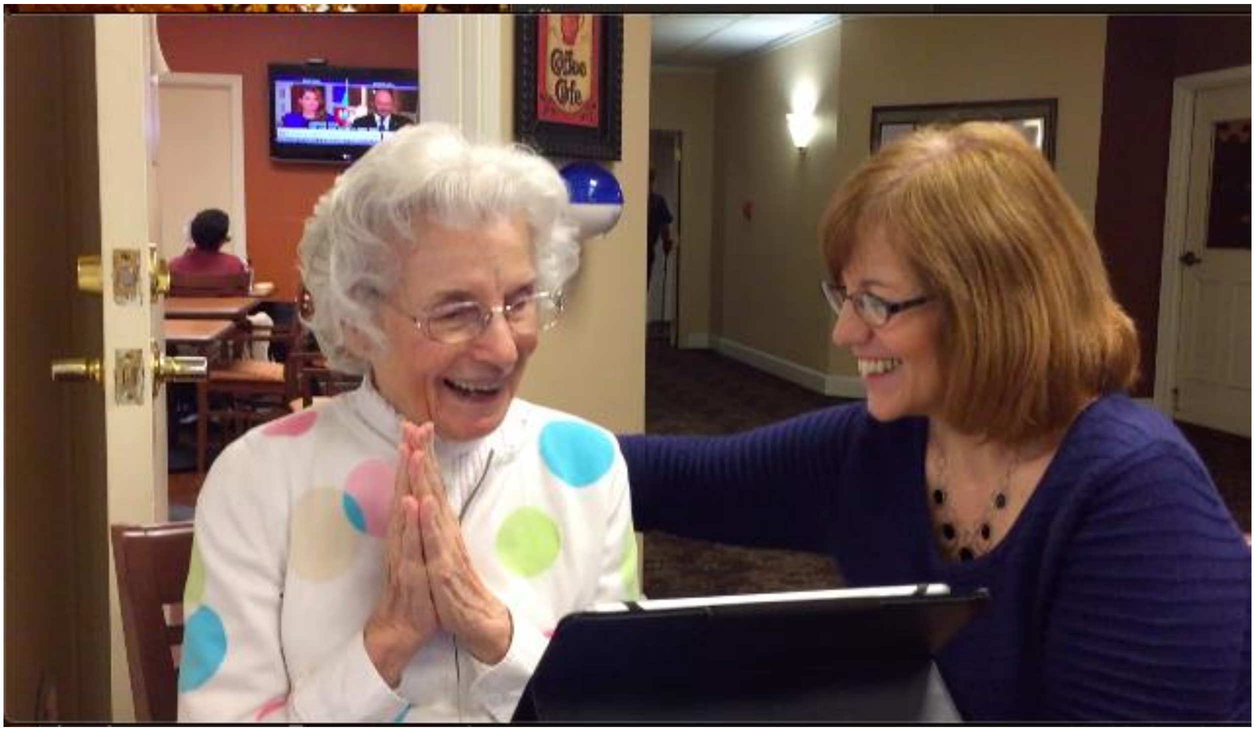

This picture demonstrates joyful mood behavior, including large smiles and physical reaction.

- "Played Scotland video, resident remembered friends but couldn't elaborate, remembers loving Scotland and has fond memories."
- "We toured Scotland today after hearing more stories of their heritage, seeing Scotland Yard, churches and of course castles."
- "Became very alert when I played the Scottish music video, really liked it"

Engagement

One care recipient had been diagnosed with dementia, a movement disorder, and aphasia; he was extremely fidgety. Through the discovery process the care team learned that he had been a manager at a large earthmoving manufacturing company. To reconnect this former manager with earlier, happier work-life experiences, the caregiver found a video of 1960s earthmoving equipment and suggested they view it together.

The feedback on the care recipient's reactions was as follows:

- *"YouTubed some earthmover videos and client was just drawn right to the screen he wouldn't look away! At one point I was going to shut it off and he grabbed the edge. He wanted to see more. We watched clips for about 30 minutes. I will definitely use this again when he is fidgety or just to entertain him."*

This study did not provide official tracking of session lengths or session participants. It tracked session feedback with staff and caregivers, and supportive interactions from an average of 15 minutes to more than an hour. Submitting feedback after a tablet session typically took less than two minutes.

Passive music listening sessions could last almost an hour when music was used as comfort:

- *"Used headphones and played carpenters concert [46.16], he loved it."*

In contrast, a session of funny cat videos might last 10 minutes:

- *"Spent 10 mins. With participant for 1:1, she's a cat lover. I pulled up weird cats on YouTube. She took the iPad from me and held it the whole time. Great smiles gotten."*

Planning

Feedback from the engagement sessions tracked the most impactful experiences and allowed caregivers to target the most meaningful music, reminiscent subject matter, and effective tablet-based activities to enhance existing routines.

Suggesting Relaxation in a Care Plan:

- "Prior to naps, during the evening, and for hospital emergencies, care teams can use the tablet with personalized activities such as reading prayers, watching nature videos, or listening to calming music to help [the individual] relax."

Using Relaxation in the Care Plan:

- A care recipient had medical problems and wound up at the emergency room for several hours one evening. As they left, the caregiver grabbed her iPad. She knew that the ER would be stressful for her client. Using the iPad the caregiver was able to provide personalized and comforting music; the care recipient stayed relaxed and interactive while in the ER.

Feedback About the Intervention:

- *"So as his daughter and I were ushered back to him, I got out the tablet and put on some of his music. His fingers immediately began tapping to the music it kept him relaxed and his mind off of the monitors that he was hooked up to. This was a successful day with music."*

Best Practices

Best practices information for this project covered three specific areas; apps, engagement strategies, and device safety and security.

Best practices for apps included but were not limited to:
- Need for Wi-Fi connection versus and apps that could be used offline by downloading information.
- Tips for deciding which care recipients would benefit from specific app categories. For example, quilters, artists and gardeners may enjoy working with colors in the Colorfy adult coloring app.
- Preload videos or games, skip through ads and double check volume prior to inviting the resident to engage.

Best practices for engagement strategies included but were not limited to:
- Psychosocial approaches.
- Introducing the activity, not the device.
- Providing choice for the participant.
- Determining the appropriate pace.
- Speaking in the line of sight.
- Assisting with a hand-under-hand technique if help needed to move forward.
- Using a 'Best Friends'* approach and working together to enjoy the engagement.

Best practices for device safety and security included but were not limited to:
- Locked location for charging and storing devices.
- Hardware cases for the tablets.
- Sanitation options.
- Procedure for a missing device.

Background Discovery Handout

Tablet Engagement Series

Resident Background Brainstorming
Use this worksheet to gather searchable background information for personalized tablet engagement. Jot down what you know, chat with the resident, ask your team, check with family, review intake forms and/or look for clues in the room.
Use these notes as inspiration to search for meaningful content on the tablet. Start by exploring YouTube videos, focusing on topics that are connected to the resident's life.

Hometown
Where was the resident raised? Were there schools or other places in the community where the resident regularly spent time?
MusicCheck out the resident's iTunes playlist. Jot down favorite artists, songs, and other relevant notes about his or her music history.

Spirituality

Does the resident practice a specific faith? Any favorite hymns or scripture? Are there any spiritual or relaxation practices such as prayer, meditation, exercise, or other techniques for relaxation?
CareerHow did the resident spend his or her work life? Include professions, responsibilities, fond memories, etc.

Animals
Is the resident an animal lover? Add notes regarding favorite animals or pets. Include breed and color if possible.

Hobbies
It is fun to relive cherished pastimes. What are some favorite interests - sports, art, quilting, gardening, fishing, card games, etc.?

Travel
Did the resident travel? Add notes regarding favorite places, travel destinations, memorable trips, etc.

Family
Add notes regarding family relationships, including parents, siblings, children, grandchildren, etc. Are there any favorite stories?
