# Supplementary material for: Using Computer Tablets to Improve Moods for Older Adults With Dementia and Interactions With Their Caregivers: Pilot Intervention Study
Source: JMIR Form Res. 2019 Sep 3;3(3):e14530. doi: 10.2196/14530 (PMC6751094; doi:10.2196/14530)
Supplement: Multimedia Appendix 3 [file formative_v3i3e14530_app3.pdf]

Appendix 3. Description of the apps included on the tablet

Apps that were available	***App Description***
4 pictures/1 word	Visual word guessing game. Four images to guess one word.
Activity Connection	Activity Department Service
Adobe Spark Video	Visual story creation app.
Animal App	No specific info given on app.
Chain of thought	A word association game that is easily played with a partner.
E-books	Download books
Elevate	Brain training app.
Email	Email communication
Facebook	Social networking
FaceTime	Video calls
Family Tree	Genealogy tracking app
Flash cards in Korean	Card game app
Flow free	Puzzle app
Garage Band	Music creation app
Geo Guesser	Location game
Google Arts & Culture	Art experience app/
Google Duo	Video call app.
Google Earth	Geolocation app.
Google Search	Web search app
Instagram	Social photo sharing app.
Jigsaw Puzzle	Traditional jigsaw puzzle app
Mahjong	App of traditional tile game
Multi-player Uno	An online multi -player version of Uno card game
News App	Access to a variety of new
Noodle Doodle	Humorous captions for doodles
Personal photos	Photos

Personal playlist	Using music services or buying music to curate personal list
Personal Video	Recorded videos from family
Pottery	Provides the ability to create pottery pieces on screen
Podcast	News and serial informational services
Recolor	Adult coloring app
Relax Meditation	Allows user to curate sounds of nature, music, etc.
Solitaire	App version of the traditional card game
Sudoku	App version of the traditional puzzle game
Tangram	Real pieces reflected to screen for puzzle
Unblock Me	Puzzle game
Virtual Exercise App	Landscape tours
Wallpaper App	On-screen art app
Web Out Loud	Website reader app
Word Search	Traditional word search game app
YouTube	Web-based video sharing app
