# Supplementary material for: Using Computer Tablets to Improve Moods for Older Adults With Dementia and Interactions With Their Caregivers: Pilot Intervention Study
Source: JMIR Form Res. 2019 Sep 3;3(3):e14530. doi: 10.2196/14530 (PMC6751094; doi:10.2196/14530)
Supplement: Multimedia Appendix 4 [file formative_v3i3e14530_app4.pdf]

Appendix 4: Feedback Survey Questions

A sample survey was demonstrated and all questions on the survey were answered using voice-to-text, or text-only functions. Staff had the opportunity to ask questions during the trainings, via phone, or email.

The feedback survey was comprised of 5 questions

- Mood prior to session? *Joyful, Happy, Relaxed, Indifferent, Sad, Anxious, Angry*
- What activity did you do?
- Briefly describe the activity. What was successful? What was challenging?
- Mood after session? *Joyful, Happy, Relaxed, Indifferent, Sad, Anxious, Angry*
- How did the engagement session impact your day?

The face scale was generally explained as follows:

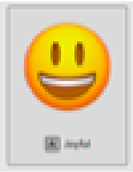

Joyful = Physical and emotional happiness present, large smiles, gesticulating, or laughter.

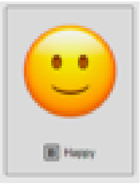

Happy = Physical comfort present, pleasant upturned smiles, emotionally merry, or cheerful.

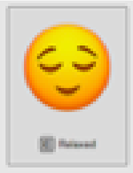

Relaxed = Physical comfort and smiles present, emotionally calm, tranquil, serene.

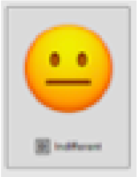

Indifferent = Physically and emotionally disinterested, lack of expression, impartial or uninvolved.

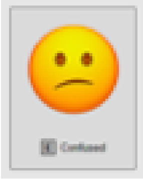

Confused = Physically unfocused, emotionally distracted, mixed-up or disconcerted.

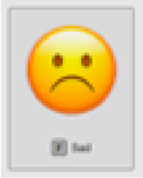

Sad = Physically slight frown, emotionally sorrowful, dejected, or troubled.

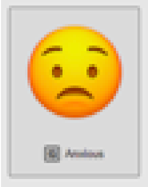

Anxious = Physically restless, emotional distress, apprehensive or antsy.

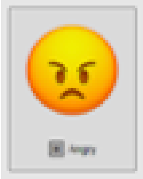

Angry = Physically Irritated, emotionally sharp, cross, inflamed.
