# Supplementary material for: Using Computer Tablets to Improve Moods for Older Adults With Dementia and Interactions With Their Caregivers: Pilot Intervention Study
Source: JMIR Form Res. 2019 Sep 3;3(3):e14530. doi: 10.2196/14530 (PMC6751094; doi:10.2196/14530)
Supplement: Multimedia Appendix 5 [file formative_v3i3e14530_app5.pdf]

Appendix 5 Frequency of Study variables.

Variables		Full sample (n=1089)	Skilled nursing facilities (n=373)	Home care (n=666)	*Other* care (n=50)
		Frequency (%)	Frequency (%)	Frequency (%)	Frequency (%)
**Primary challenge**					
	Memory loss	785 (72.08)	165 (44.2)	582 (87.4)	38 (76)
	Non-ambulatory	105 (9.64)	102 (27.3)	0 (0)	3 (6)
	Movement disorder	128 (11.75)	41 (11.0)	84 (12.6)	3 (6)
	Communicative or expressive disorders	56 (5.14)	55 (14.7)	0 (0)	1 (2)
	Other	15 (1.38)	10 (2.7)	0 (0)	5 (10)
**Engagement method used (singularly or one of multiple)**					
	Music	377 (34.62)	115 (30.8)	253 (38.0)	9 (18)
	Stories video	199 (18.27)	84 (22.5)	103 (15.5)	12 (24)
	Games	113 (10.38)	33 (8.8)	65 (9.8)	15 (30)
	Communication	96 (8.82)	48 (12.9)	48 (7.2)	0 (0)
	Photos	75 (6.89)	16 (4.3)	56 (8.4)	3 (6)
	Other	86 (7.90)	66 (17.7)	10 (1.5)	10 (20)
	Missing	143 (13.13)	11 (2.9)	131 (19.7)	1 (2)
**Type of strategies used**					

**Music**[a]				
Singularly	199 (38.8)	61 (49.6)	135 (35.4)	3 (33)
One of multiple	314 (61.2)	62 (50.4)	246 (64.6)	6 (67)
**Reminiscing**[b]				
Singularly	43 (14.1)	37 (27.4)	4 (2.6)	2 (13)
One of multiple	261 (85.9)	98 (72.6)	150 (97.4)	13 (87)
**Socialization**[c]				
Singularly	186 (34.7)	62 (32.6)	119 (36.8)	5 (22)
One of multiple	350 (65.3)	128 (67.4)	204 (63.2)	18 (78)
**Relaxation**[d]				
Singularly	13 (5.5)	8 (29)	2 (1.0)	3 (50)
One of multiple	223 (94.5)	20 (71)	200 (99.0)	3 (50)
**Achievement**[e]				
Singularly	103 (41.0)	41 (59)	48 (29.3)	14 (78)
One of multiple	148 (59.0)	28 (41)	116 (70.7)	4 (22)
**Number of strategies used**				
1	541 (49.68)	206 (55.2)	308 (46.2)	27 (54)
≥2	525 (48.21)	158 (42.4)	346 (52.0)	21 (42)
Missing	23 (2.11)	9 (2.4)	12 (1.8)	2 (4)
**Type of app used for strategies**				
YouTube only	305 (28.01)	167 (44.8)	122 (18.3)	16 (32)
Personal Playlist only	308 (28.28)	5 (1.3)	303 (45.5)	0 (0)
Personal photos or videos only	76 (6.98)	36 (9.7)	39 (5.9)	1 (2)
Google only	91 (8.36)	40 (10.7)	46 (6.9)	5 (10)
Puzzle only	78 (7.16)	28 (7.5)	41 (6.2)	9 (18)
Combinations of apps	107 (9.83)	24 (6.4)	75 (11.3)	8 (16)

	Other	101 (9.27)	63 (16.9)	27 (4.1)	11 (22)
	Missing	23 (2.11)	10 (2.7)	13 (2.0)	0 (0)
**Number of apps used for strategy**					
	1	958 (87.80)	339 (90.9)	577 (86.6)	42 (84)
	≥2	107 (9.83)	24 (6.4)	75 (11.3)	8 (16)
	Missing	24 (2.20)	10 (2.7)	14 (2.1)	0 (0)
**Urban or rural status**					
	Rural	409 (37.56)	270 (72.4)	121 (18.2)	18 (36)
	Urban	680 (62.44)	103 (27.6)	545 (81.8)	32 (64)
**Facility type**					
	Skilled nursing	373 (34.25)	373 (100.0)	0 (0)	0 (0)
	Home care	666 (61.16)	0 (0)	666 (100.0)	0 (0)
	Other	50 (4.59)	0 (0)	0 (0)	50 (100)
**Mood change**					
	Worsening of mood	8 (0.73)	2 (0.5)	6 (0.9)	0 (0)
	Maintaining negative mood	72 (6.61)	26 (7.0)	40 (6.0)	6 (12)
	Maintaining positive mood	456 (41.87)	42 (11.3)	393 (59.0)	21 (42)
	Improvement of mood	553 (50.78)	303 (81.2)	227 (34.1)	23 (46)
**Session impact on caregiver's daily activities[f]**					
	1, made it worse	0 (0)	0 (0)	0 (0)	0 (0)
	2	0 (0)	0 (0)	0 (0)	0 (0)
	3, no impact	41 (7.6)	4 (12)	36 (7.4)	1 (2)
	4	322 (59.4)	20 (61)	294 (60.6)	8 (16)
	5, made it better	179 (33.0)	9 (27)	155 (32.0)	15 (30)
	Missing	0 (0)	0 (0)	0 (0)	26 (52)

a. Given that not all care recipients used music as a strategy, the sample size for this variable varied depending on where care was received. The actual sample sizes were 513 (Full Sample), 133 (Skilled Nursing), 381 (Home care) and 9 (Other)

b. Given that not all care recipients used reminiscing as a strategy, the sample size for this variable varied depending on where care was received. The actual sample sizes were 304 (Full Sample), 135 (Skilled Nursing), 154 (Home care) and 15 (Other).

c. Given that not all care recipients used socialization as a strategy, the sample size for this variable varied depending on where care was received. The actual sample sizes were 536 (Full Sample), 190 (Skilled Nursing), 323 (Home care) and 23 (Other).

d. Given that not all care recipients used relaxation as a strategy, the sample size for this variable varied depending on where care was received. The actual sample sizes were 236 (Full Sample), 28 (Skilled Nursing), 202 (Home care) and 6 (Other).

e. Given that not all care recipients used achievement as a strategy, the sample size for this variable varied depending on where care was received. The actual sample sizes were 251 (Full Sample), 69 (Skilled Nursing), 164 (Home care) and 18 (Other).

f. Given that caregiver daily activities were not measured in every facility, the sample size for this variable varied depending on where care was received. The actual sample sizes were 542 (Full Sample), 33 (Skilled Nursing), 485 (Home care) and 50 (Other).
